# myoActivation®, a Structured Assessment and Therapeutic Process for Adolescents With Myofascial Dysfunction and Chronic Low Back Pain: A Case Series

**DOI:** 10.7759/cureus.68029

**Published:** 2024-08-28

**Authors:** Jessica Luo, Nicholas West, Gillian R Lauder

**Affiliations:** 1 Department of Pediatric Anesthesia, British Columbia Children’s Hospital Research Institute, Vancouver, CAN; 2 Faculty of Science, University of British Columbia, Vancouver, CAN; 3 Department of Anesthesiology, Pharmacology, and Therapeutics, British Columbia Children's Hospital Research Institute, Vancouver, CAN

**Keywords:** case series, interdisciplinary pain clinic, myofascial dysfunction, chronic pain, low back pain

## Abstract

Introduction

Myofascial dysfunction is a significant, but often unrecognized, contributor to chronic low back pain (CLBP). myoActivation is an innovative method that utilizes a structured assessment and therapeutic process to recognize and manage myofascial dysfunction and pain. Since 2017, the British Columbia Children’s Hospital Complex Pain Service has used myoActivation as a tool in the interdisciplinary care of adolescents with chronic pain. This case series explores the journey to discharge of patients in whom myoActivation was incorporated as part of their CLBP management.

Methods

We retrospectively reviewed clinical records of adolescents reporting CLBP who underwent myoActivation between August 2022 and January 2023 and had subsequently been discharged. Information obtained for analysis included preclinical information (medical/injury/pain history, previous investigations, diagnoses, therapies, and quality of life indicators); clinic recommendations, assessment findings, management strategies, and specifics of the myoActivation process; and reported changes at discharge (quality-of-life measures and medication use).

Results

Eight cases were reviewed: all female, with a median age (range) of 16.5 (15.7-19.5) years. Before admission, patients had experienced chronic pain for a median duration of 4.3 (1-8) years, had self-reported average pain intensity of 7.5 (4-9) on the 0-10 numeric pain scale, with poor quality-of-life impacts including sleep disturbance (8/8, 100%), school absence (8/8, 100%), and low mood (6/8, 75%). Patients attended three (2-5) myoActivation sessions over two (1-10) weeks. The overall duration of their interdisciplinary care was 12 (7-25) months. At discharge, there were improvements in pain (7/8, 88%), physical functioning (5/8, 63%), sleep (6/8, 75%), school attendance (5/8, 63%), and mood (4/6, 67%) and reduced prescription and over-the-counter medication use in most cases.

Conclusion

This case series suggests that myoActivation may be a useful clinical tool in the assessment and management of adolescents with myofascial dysfunction and CLBP. Prospective longitudinal research is required to establish evidence that confirms the clinical efficacy of myoActivation within interdisciplinary care.

## Introduction

Chronic low back pain (CLBP), described as pain that persists for over three months in the lumbar region [1], is a common and disabling health issue worldwide [2]. CLBP is not isolated to the adult population: 49% of high school students aged 14-19 years old experience one or more low back pain episodes [3]. Ongoing chronic pain in adolescents can have debilitating consequences for physical, psychological, and social functioning [4] with an associated loss of mobility, inability to participate in physical activities, decreased sleep quality, difficulty concentrating on schoolwork, school absenteeism, social isolation, and family stress [5,6]. Anxiety and depression are also known to be highly comorbid in people with a lived experience of chronic pain [7]. Non-specific CLBP [8], where a known remedial cause for pain has been excluded, can impact day-to-day functioning in up to 13% of adolescents [3]. This leaves them not only with unresolved pain and suffering but also with diagnostic uncertainty. These patients are often discharged from subspecialty care and advised to seek allied health alternatives, such as physiotherapy or psychology support. Individuals with chronic pain may experience stigma, be dismissed by peers or medical providers, and are often given the impression that the pain is “all in their head” [9]. Overlooked causes for pain and inadequate pain management may result in ongoing painful movements, fear of movement (kinesiophobia), a deconditioned physical status, illness-related distress, poor psychological functioning, and/or a general mistrust of the medical system [10].

Myofascial dysfunction and pain

Myofascial dysfunction is a significant yet often unrecognized contributor to CLBP in the pediatric population [11]. Myofascial dysfunction and pain (MFD&P) can be diagnosed by identifying postural anomalies, muscle trigger points, scars, fascia in tension, or a combination of these findings. Trigger points (palpable pain points) can exist in muscles and fascia. Muscular trigger points are irritable nodules, located predominantly near the motor end plates in taut bands of skeletal muscle [12], which can develop in response to trauma, injury, surgery, repetitive microtrauma, poor posture, muscle overuse, or overload [13,14]. Fascial trigger points are palpable painful densities, often located near active muscle trigger points. Scars also contribute to defective fascial sliding, leading to distorted biomechanics and chronic pain. Scars are common in the pediatric population and are significant contributing factors to MFD&P, even when they appear to look “normal” [15].

The 3P conservative interdisciplinary approach

The Complex Pain Service (CPS) at British Columbia Children’s Hospital provides assessment and management to pediatric patients with chronic and/or complex pain. Patients are referred to the CPS by a healthcare provider, family doctor, nurse practitioner, pediatrician, or subspecialty physician; patients are discharged from CPS when they meet their functional goals. The CPS utilizes an interdisciplinary pain care approach, which is now considered a standard of care for pediatric complex pain. This approach is more effective than single-discipline treatments or outpatient non-interdisciplinary rehabilitation [16]. Conservative management includes the 3P (physiotherapy, psychology, and pharmacology) approach, designed to help youth living with chronic pain achieve the goal of changing from a pain-centered life to a function-centered life. All CPS patients, regardless of their pain location and presentation, are initially managed with this 3P approach. In addition to seeking support from a physiotherapist, psychologist/therapist, and pharmacologist, patients diagnosed with MFD&P are also advised to engage in massage therapy and optimize supplements (magnesium bis-glycinate, vitamin K2, vitamin D3, and omega 3) designed to promote relaxation of muscles in sustained contraction and support an anti-inflammatory environment [11]. These conservative measures are trialed for four to six weeks. If a patient’s pain improves with initial conservative management such that functional goals can be achieved, then no added interventions are required. If repeat examination after this period reveals that there is still a myofascial component to pain, appropriately selected patients are offered myoActivation. Written information about MFD&P and myoActivation is given to the patient and family at this time.

Objectives

In this case series, we explore the journey from CPS admission to discharge of patients in whom myoActivation was incorporated as part of their CLBP management. The purpose of this case series is to 1) illustrate the utility of the myoActivation assessment to diagnose a myofascial component in CLBP, 2) identify some of the myofascial tissues likely contributing to MFD&P for adolescent CLBP, and 3) provide preliminary data of myoActivation therapy as a useful tool in adolescent CLBP multidisciplinary management. These study findings were previously presented as a meeting abstract at the 2024 Society for Pediatric Pain Management (SPPM) Annual Scientific Meeting on April 11, 2024.

## Materials and methods

We retrospectively reviewed clinical records of adolescents aged 13-19 years old within the CPS. Patients were included if they reported CLBP and underwent myoActivation between August 2022 and January 2023. Patients were excluded if they had not yet been subsequently discharged from the CPS. The University of British Columbia - Children’s & Women’s Health Centre of BC Research Ethics Board waived the requirement for individual consent in approving this retrospective study (H21-00951, 02-Jun-2021).

Key features of the patients’ conditions, treatment techniques, and patients’ responses were extracted and described. Information was gathered from the referring physician’s referral letter, completed parent and child admission forms, and documentation from patient appointments with the CPS. Information extracted from these records, when available, included (i) demographics; (ii) preclinical information, such as pain, trauma history, medical history, previous investigations, diagnoses, medical and other therapies, goals of CPS care, and the impact of pain on function and quality of life; (iii) details of CPS care, such as clinic recommendations, assessment, and interventions, and details of myoActivation assessment and therapeutic interventions; and (iv) discharge information, including recorded improvements at discharge for pain, sleep, mood, physical function, school attendance and goals of CPS care. Microsoft Excel (Microsoft Corporation, USA) was used for data collection and descriptive analysis.

Assessment and treatment approach: myoActivation®

myoActivation® is an innovative, structured approach designed to resolve MFD&P [17] (see Intellectual Property: Patents & Copyrights). This process, described in detail below, employs (i) a unique injury history, known as the Timeline of Lifetime Trauma (TiLT), (ii) a structured assessment process, and (iii) a non-pharmacological, needling therapy. myoActivation treatment results in immediately observed and reported changes in pain, flexibility, and ease of movement [18]. myoActivation combines three well-established techniques for managing chronic pain: intramuscular stimulation, scar release, and fascial release. The needling component of myoActivation is much less invasive than acupuncture, which is also an established technique for the management of chronic pain, including in children. For appropriately selected patients, myoActivation is discussed during their appointments, and consent is obtained from both the primary caregiver and youth. myoActivation is not offered to youth with needle aversion or procedural anxiety.

Timeline of Lifetime Trauma (TiLT)

The TiLT is a unique, detailed injury history focusing on past injuries, fractures, surgeries, and the associated scars as far back as the patient and family can remember. The most significant contributor to MFD&P is often the injury or scar from the youngest age. The mechanism of injury is sought for each event to help elucidate which soft tissues may have been impacted by an injury.

Structured Posture and Movement Assessment

**Figure 1:**
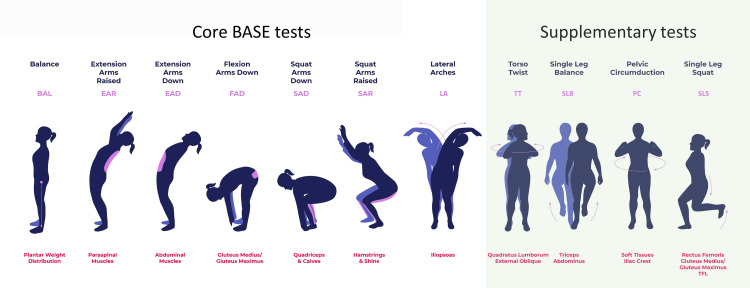
Biomechanical Assessment and Symmetry Evaluation (BASE) tests. The seven core tests include (left to right): balance (BAL), extension arms raised (EAR), extension arms down (EAD), flexion arms down (FAD), squat arms down (SAD), squat arms raised (SAR), and lateral arches (LA). The related tissues are described below each movement demonstration, in red text. Originally published by Bhatnagar *et al.* [17]. Image credits: Gillian R. Lauder with illustrations by Shona Massey

myoActivation assesses for postural anomalies and limitations in systemized movement tests. Posture assessment includes the determination of the patients' perceived weight on feet, head position, shoulder height, torso alignment, hip height, pelvic orientation, and knee and feet positioning; all as indicators of differences in the symmetry of the body. The standardized movements are known as Biomechanical Assessment and Symmetry Evaluation (BASE) tests (Figure 1). The most restricted or painful BASE test indicates to the myoActivation clinician the most likely area where soft tissues are impacting myofascial dysfunction. For example, if the most restricted or painful BASE test is extension arms down (EAD), then the tissues most likely to be impactful are the abdominal wall tissues. The myoActivation clinician would inspect and palpate the abdominal wall to determine if there were muscles in sustained contraction, fascia in tension, or scars. The information amalgamated from the TiLT, posture assessment, and the examination help to determine the important soft tissue or tissues (as there may be more than one area contributing) to each individual patient's MFD&P.

Treatment: Non-pharmacological Needling Technique

myoActivation intervention is a needling technique utilizing fine gauge hollow-bore, cutting-tip hypodermic needles to release; significant scars, muscles in sustained contraction (known as activation), and fascia in tension [15,17]. To prevent needle-related pain in pediatric patients, the clinician should always employ a variety of non-pharmacological techniques including distraction, modification of breathing techniques, music, virtual reality, or mobile devices. A topical anesthetic cream can be applied to the target sites (especially scars) an hour before the appointment to minimize needle pain. Vapocoolant spray is also a useful topical agent to minimize discomfort with needling. Electrical stimulation is not used for myoActivation. The BASE tests and palpation are repeated after each treatment to determine the next most dominant myofascial soft tissue(s) that need to be treated. This repeated treatment and assessment process is known as "catenated cycles" [17]. The number of catenated cycles in any one myoActivation session depends on individual treatment needs, tolerance, and response. Once the BASE tests are improved, the myoActivation clinician uses different regional tests to resolve other sources of myofascial dysfunction, such as neck pain or headaches. myoActivation sessions are usually spaced one to two weeks apart. Patients may require two to six sessions to completely unravel multiple sources of MFD&P.

Side effects include discomfort at the site of needling, infection, bruising, light-headedness, post-care fatigue, muscle aches, emotional responses, and, extremely rarely, pneumothorax when needling around the lung fields.

## Results

This retrospective case series explores the journey to discharge of eight pediatric patients with MFD&P and CLBP who received myoActivation as a part of their interdisciplinary care with the CPS. Their median (range) age was 16.5 years (15.7-19.4) with a weight of 57.9 (46.5-73.6) kg, and they presented with a range of diagnoses, comorbidities, and specific pain locations (Table 1).

**Table 1:** Patient demographics. kg: kilograms, y: years, m: months

ID	Demographics			Specialty of the referring physician	Referral medical diagnosis and comorbidities	Locations of pain
	Age	Weight (kg)	Sex			
1	16 y, 1 m	76.2	Female	Pediatrician	Endometriosis and associated chronic pain, vasovagal syncopes, trigger: pain, chronic gastrointestinal symptoms, elevated body mass index, anxiety, depression	Low back, legs, and feet
2	16 y, 5 m	51.9	Female	Orthopedics	Scoliosis, anxiety, panic attacks, attention-deficit/hyperactivity disorder, self-harm	Low back
3	16 y, 5 m	63	Female	General Practice/Family Medicine	Ehlers-Danlos syndrome, asthma, fatigue, attention-deficit/hyperactivity disorder, autism spectrum disorder (high-functioning), developmental coordination disorder, learning disorder	Low back
4	18 y 8 m	46.5	Female	Psychiatry	Unspecified anxiety disorder + post-traumatic stress disorder, panic attacks, unspecified mood disorder	Low back to feet
5	16 y 11 m	60.5	Female	Gynecologist	Recurrent urinary tract infections, recurrent bladder stones, ovarian cysts, specific learning disorders in reading, writing, and math, language disorders in both expressive and receptive skills	Low back to central back
6	15 y 8 m	53.8	Female	Orthopedics	Scoliosis (40 degrees), multisite pain, anxiety, and depression	Low back, upper back, and neck
7	17 y 3 m	55.3	Female	Pediatrician	Herniated lumbar disc (non-surgical) and moderate developmental narrowing of the lumbar canal	Low back and legs
8	19 y, 5 m	73.6	Female	Orthopedics	Multilevel spondylolysis (no spondylolisthesis) and anxiety	Low back, hips, and upper back

Before the CPS admission, the patients had experienced chronic pain for a median (range) of 4.3 (1-8) years with a self-reported average pain intensity of 7.5 (4-9) out of 10. All patients had undergone medical investigations to exclude a remedial cause of chronic pain. Prior to the CPS assessment, many had trialed various pharmacological and non-pharmacological techniques for their pain, such as prescribed medications, over-the-counter medications, massage therapy, physiotherapy, chiropractic treatments, or acupuncture (Table 2). The patients were referred to the CPS between March 2020 and December 2021, in the first two years of the COVID-19 pandemic.

**Table 2:** Investigations and treatments for pain, prior to CPS admission. CPS: complex pain service, MRI: magnetic resonance imaging

ID	Investigations	Pharmacological treatments	Non-pharmacological treatments
1	Bloodwork, X-Ray	Cannabidiol oil, citalopram, gabapentin, ibuprofen/acetaminophen combined	Acupuncture, chiropractic, counseling/psychology, massage therapy
2	MRI, scoliosis clinic, X-ray	Cannabidiol oil, fluoxetine, ibuprofen	Counseling/psychology, massage therapy, physiotherapy, yoga
3	Rheumatology, X-ray	Acetaminophen, ibuprofen	Chiropractic, meditation, physiotherapy, transcutaneous electrical nerve stimulation machine
4	Adolescent medicine, bone scans, MRI, neurology, psychiatry, rheumatology, X-rays	Amitriptyline, duloxetine, fluvoxamine, gabapentin, mirtazapine, pregabalin	Chiropractic, counselling/psychology, massage therapy, physiotherapy
5	MRI, ultrasound, X-rays	Acetaminophen, ibuprofen	Not documented
6	Scoliosis clinic, X-ray	Acetaminophen, diclofenac	Chiropractic, massage therapy, physiotherapy
7	MRI, X-ray	Gabapentin, ibuprofen	Massage therapy
8	MRI, X-rays	Pregabalin	Chiropractic, counseling/psychology, craniosacral therapy, intramuscular stimulation dry needling

The majority of patient histories revealed previous physical trauma (surgery, fracture, or fall on the coccyx) (Table 3), and all were taking prescribed or over-the-counter medications for pain at the time of their initial assessment (Table 4). All experienced negative impacts on their quality of life: sleep disturbance in 8/8 (100%), school absence in 8/8 (100%), and low mood in 6/8 (75%). All patients were asked to identify their specific treatment goals at their initial assessment with the CPS. Each patient had different self-reported explanations for pain, including “endometriosis” (Patient 1), “scoliosis surgery” (Patient 2), “Ehlers-Danlos syndrome” (Patient 3), “somatic symptom disorder and post-traumatic stress disorder” (Patient 4), and spine-related conditions, like “prolapse disk” (Patient 5), “scoliosis” (Patient 6), “underdeveloped lumbar, bulging (oversized) disk impeding on nerve system, narrow spinal column with lower than average spinal fluid” (Patient 7), or “spondylolysis and obsessive-compulsive disorder” (Patient 8).

**Table 3:** Patient Timeline of Lifetime Trauma (TiLT).

ID	Previous fractures	Previous surgeries	Previous fall on the coccyx	Scars; self-identified by the patient
1	None	None	None	Right groin, left hand, and bilateral knee scars
2	None	Double spinal tethering for idiopathic scoliosis	None	5x right chest wall, and 2x left flank
3	Dislocation of knee and ribs	None	Yes	Stretch marks along the anterior iliac crest
4	Grade 5 - spiral tibia + fibula break	None	None	Back of the right hand, right bicep, right knee, left arm; healed self-harm scar
5	Right elbow dislocation and fracture	Radical cystectomy	None	Bilateral upper chest/collarbone, abdominal, bilateral and midline umbilicus
6	None	None	Yes	None
7	None	None	Yes	None
8	Right arm	Anterior cruciate ligament reconstruction (bilateral), tonsillectomy and myringotomy	Yes	Bilateral knee scars, left shoulder

**Table 4:** Medications at Complex Pain Service admission and discharge. BID: bis in die/twice daily, PRN: pro re nata/as needed, QD: quaque die/once daily Patients may have some missing discharge information as clinical record-keeping focused on important aspects and some questions have been omitted by their healthcare provider at the time.

ID	Timepoint	Prescribed	Over the counter	Supplements	Non-medical
1	Admission	Citalopram, gabapentin, oral contraceptive pill	Acetaminophen, ibuprofen, melatonin	Not documented	Cannabidiol oil
	Discharge	Not documented	Not documented	Magnesium bisglycinate, omega-3, vitamin D3, vitamin K2	Not documented
2	Admission	Acetaminophen with methocarbamol (BID), sertraline	Acetaminophen, ibuprofen	Not documented	None
	Discharge	Methylphenidate (PRN), pregabalin (QD), sertraline	None	None	None
3	Admission	Fluticasone propionate, salbutamol	Acetaminophen	Not documented	Not documented
	Discharge	Acetaminophen with methocarbamol, fluticasone propionate, lisdexamfetamine dimesylate, salbutamol (PRN)	Acetaminophen (rarely), ibuprofen (rarely)	None	Alcohol
4	Admission	Amitriptyline, fluvoxamine, oral contraceptive implant	Not documented	Ashwagandha root extract and L-theanine, elemental iron, vitamin D	Cannabis
	Discharge	Compound lidocaine 5% and diclofenac 15% in lipoderm, lemborexant, pantoprazole, quetiapine (PRN)	Not documented	Magnesium bisglycinate, omega-3, vitamin D3, vitamin K2	Not documented
5	Admission	Nitrofurantoin (one week), trimethoprim/sulfamethoxazole (one week)	Not documented	Not documented	Alcohol, Vape (nicotine content unknown)
	Discharge	None	None	Magnesium bisglycinate, omega-3, vitamin D3, vitamin K2	Not documented
6	Admission	Doxycycline (for sinusitis), oral contraceptive pill, pantoprazole, sertraline	Acetaminophen, ibuprofen	Not documented	Not documented
	Discharge	Not documented. Oral contraceptive pill, sertraline	Not documented	Not documented	Not documented
7	Admission	Pregabalin	Ibuprofen	Elemental iron	None
	Discharge	None	None	Vitamin D3, zinc	Topical cremes
8	Admission	Isotretinoin, naproxen, tylenol-3 (does not use)	None	Magnesium, selenium, zinc	Not documented
	Discharge	Duloxetine	Codeine/acetaminophen (PRN)	Magnesium bisglycinate, omega-3, vitamin D3, vitamin K2	Not documented

Based on their initial myoActivation assessment findings, including initial BASE test results, combined with their TiLT (Table 3), pain history, medical history, and classical examination, all cases were diagnosed with a myofascial component to their pain (Table 5). Patients were given education and written information about MFD&P at the time of their diagnosis. The most common co-morbid diagnoses were anxiety disorders (generalized anxiety disorder, panic attacks, and/or obsessive-compulsive disorder) (Table 5).

**Table 5:** myoActivation assessment. BASE: Biomechanical Assessment and Symmetry Evaluation, ROM: range of motion, CPS: Complex Pain Service

ID	Initial reported weight on feet	Initial worst BASE test for pain	Initial worst BASE test for ROM	Other scars; previously unidentified by the patient	Diagnosis on the initial CPS assessment
1	Right/lateral/back	Lateral arch	Flexion arms down	Right forearm scars and right popliteal fossa scars	Chronic low back pain & myofascial dysfunction, endometriosis
2	Left/medial/middle	Not documented	Lateral arch – going right	None	Chronic low back pain & myofascial dysfunction, anxiety, panic attacks, attention deficit hyperactivity disorder, self-harm
3	Left/central/back	Not documented	Not documented	Scar on the left shoulder and left arm, left lateral thigh, left knee, left shoulder, right inner thigh	Chronic low back pain & myofascial dysfunction, asthma, attention deficit hyperactivity disorder, autism spectrum disorder, ehlers danlos syndrome, fatigue, somatic symptom disorder
4	50-50/central/ front	Extension arms down	Squat arms down	None	Chronic low back pain & myofascial dysfunction, anxiety, panic attacks, avoidant-restrictive food intake disorder, obsessive compulsive disorder, post-traumatic stress disorder (history of sexual assault & self-harm), somatic symptom disorder
5	Right/lateral/back	Extension arms raised	Extension arms raised	None	Chronic low back pain & myofascial dysfunction
6	Left/lateral/back	Extension arms down	Extension arms raised and lateral arch – going left	Acne scars on the front of the chest, sternal notch scars	Chronic low back pain & myofascial dysfunction, anxiety, depression, iron-deficient, mid-back and upper back pain, scoliosis
7	Left/medial/front	Extension arms down	Flexion arms down	Acne scars on the upper and lower back	Chronic low back pain & myofascial dysfunction, disc herniation (L5-L6) with left L6 nerve root compression, headaches, lumbarization (S1 on L6 vertebrae), moderate developmental narrowing of the lumbar canal
8	Left/central/back	Extension arms raised	Extension arms down and flexion arms down	Left shin scars, chickenpox scars on the back right thigh scar	Chronic low back pain & myofascial dysfunction, anxiety, left knee pain, multilevel spondylosis, obsessive-compulsive disorder

**Figure 2:**
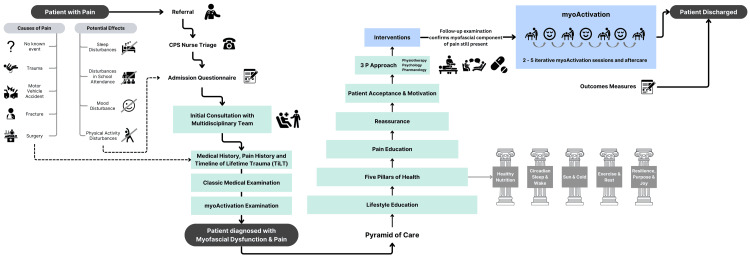
Typical flow of a patient through the CPS, including myoActivation intervention. CPS: Complex Pain Service Image credits: Jessica Luo (Created on www.canva.com)

The patients included in this series trialed a median (range) of 15 (8-83) weeks of conservative management (Figure 2). After this period, two of these patients reported feeling better, but all reported some ongoing chronic pain. myoActivation re-examination indicated a persistent myofascial component in their pain profile, so the patients were offered myoActivation.

Patients in this series attended a median (range) of three (2-5) myoActivation sessions over two (1-10) weeks. For these eight cases, the most commonly activated/released muscles were the iliopsoas (n = 7), paraspinal muscles (n = 7), abdominal wall muscles (rectus abdominus and lateral abdominal wall muscles) (n = 7), quadriceps (rectus femoris/vastus lateralis and vastus medialis) (n = 4), quadratus lumborum (n = 4), and the gluteal muscles (gluteus medius and gluteus maximus) (n = 2). The most commonly treated fascia was at the iliac crest (n = 4) and coccyx (n = 3). The most common scars released were knee scars (n = 5). All eight patients had scars released (Table 6). There were no complications of myoActivation in any of these patients.

**Table 6:** myoActivation treatment.

ID	Session	Scars	Fascia	Muscles
1	1	Iliac crest (right)	-	Iliopsoas (bilateral), paraspinals; thoracic and lumbar (right)
	2	-	-	Trapezius (bilateral), levator scapulae (right), platysma (right), paraspinals; cervical thoracic
	3	-	Pubic fascia (bilateral)	Radius abdominis (bilateral), external oblique (bilateral)
	4	Knee scars (bilateral)	-	Radius abdominis (bilateral)
2	1	Chest wall scars (bilateral)	Iliac crest (left)	Iliopsoas (left), quadratus lumborum, serratus anterior (left)
	2	Portion of one scar that was still tethered	Line of tension across the abdomen at the level of the umbilicus	Rectus abdominis (bilateral), paraspinals (bilateral), iliopsoas (right)
3	1	-	Line of tension at the subcostal margin	Rectus abdominis above the umbilicus (left), external obliques (bilateral), iliopsoas (left)
	2	Knee scars (bilateral), shoulder scar (left)	Line of tension at the subcostal margin	Tensor fasciae latae (left), vastus lateralis (left), coracobrachialis (right)
	3	-	Iliac crest (right)	Quadratus lumborum (right), tensor fasciae latae on the vastus medialis (right), serratus anterior (right and left)
	4	-	Line of tension below the subcostal margin	Paraspinals (right)
	5	-	Line of tension just above and below the umbilicus	External obliques (bilateral), quadratus lumborum (left), rectus femoris (left), vastus lateralis, vastus medialis, serratus anterior muscles (bilateral)
4	1	Low lumbar region (left)	Coccyx	Paraspinals; lumbar (bilateral), external oblique (left)
	2	-	-	Paraspinal (right), external oblique (left)
5	1	-	Coccyx	Paraspinal (right)
	2	Abdominal scars x4	-	Rectus abdominus (left)
	3	Abdominal scars	Coccyx, pubic fascia (left)	Paraspinal (bilateral)
	4	Abdominal scars	Linea alba fascia	Paraspinal (right), iliopsoas (left), vastus medialis (left)
6	1	-	-	Iliopsoas (bilateral), external oblique (right)
	2	-	Iliac crest (right)	Quadratus lumborum (right), trapezius (left and right), levator scapula (left)
	3	Shin (left), knee (bilateral)	-	Serratus anterior (right)
7	1	-	Iliopsoas (left)	Paraspinal (right), glute medius (right)
	2	Thigh (left)	Line of tension above the umbilicus	Vastus medialis (left), paraspinals; thoracic & lumbar (right)
	3	-	External oblique (left), knee (right)	Gluteus maximus (right), vastus medialis (bilateral)
8	1	Knee (bilateral)	-	Iliopsoas (left)
	2	-	Iliac crest (left and right)	Gluteus medius (left and right) and maximus (right), paraspinal (right), rectus abdominus (left)
	3	-	Iliac crest (left and right), coccyx	Quadratus lumborum (left and right)
	4	Knee scars, thigh (right), shoulder scar (right)	Sternoclavicular fascia (right)	Paraspinal muscle on the gluteus maximus (right), vastus medialis, platysma (right) on the bilateral trapezius

The median (range) time between their final myoActivation session with the CPS and discharge from the CPS was 24 (5-52) weeks. Upon discharge, changes were reported in physical and mental well-being: pain improved for 7/8 (88%), physical functioning for 5/8 (63%), sleep for 6/8 (75%), school attendance for 5/8 (63%), and mood for 4/6 (67%). Each patient reported improvement in at least one of the quality-of-life domains. Initial goals of CPS care were achieved for 7/8 (88%) patients at discharge (Table 7). Changes in medication can be found in Table 4. For the cases, the total duration of care with the CPS was 12 (7-25) months.

**Table 7:** Self-reported goals of CPS care. CPS: Complex Pain Service

ID	Patient-reported goal(s); activities they would like to do again	Achieved goals of CPS care at discharge?
1	Get my pain better under control and establish treatment options; Would like to walk normally without a cane, would like to do extracurriculars and walk again.	Yes
2	Better ways of handling my pain; “Less pain” and improved school attendance. Would most like to do “sweeping” again.	Yes
3	Reduced pain, pain fatigue, and irritability, understanding why the increase in pain as she’s gotten older; Would just like to be more physically active, without being worried about injury.	Not documented
4	Bringing my constant pain down to a more bearable level and learning to manage my feelings when the pain escalates and somehow treat that pain; Would like to attend university in the fall and be able to dance again.	Yes, except for dancing
5	To be able to complete daily tasks such as work and activities without pain and discomfort. Find what is causing her back pain which affects her walking and work; Would like to increase enjoyment in regular activities.	Yes
6	I hope to identify specific sources of my pain/discomfort and learn how to cope with it or even make it better as I know I will be dealing with it over the course of my life.	Yes
7	To be relieved of my pain and be able to participate in normal activities for people my age; Would like to not be restricted because of chronic pain.	Yes
8	Living a full life; getting rid of pain; being functional; would like to hike and work out again.	Yes

## Discussion

This case series describes the journey to discharge of eight adolescent patients with complex pain that included CLBP. We illustrate how myofascial dysfunction was a significant contributor to their pain presentation and that myoActivation was an integral part of their assessment and interdisciplinary pain management. Like many people with a lived experience of chronic pain, the adolescents in this case series had unique and complex contributing factors impacting their pain profiles. These cases had experienced a median of four years of pain prior to CPS clinic admission and had sought care from general practice or subspecialty physicians.

Interdisciplinary pain management addressing all the components of the biopsychosocial model is recognized as the gold standard of care for managing complex presentations [16,19]. Prior to CPS admission, all patients in this series trialed multiple therapeutic modalities (Figure 2). After the CPS initial assessment, they underwent a median of four months of conservative interdisciplinary pain management, incorporating a combination of physiotherapy, massage therapy, psychology, self-management strategies, and pharmaceutical or nutraceutical interventions. Despite this conservative management, our patients continued to experience chronic pain, and all were offered myoActivation. At discharge, almost every patient in this case series reported improvements in pain and achieved their self-reported initial CPS goals. myoActivation proved to be an effective tool in assessment and management when chronic pain and functioning did not improve with many other trialed modalities. The positive patient outcomes and lack of complications in this series suggest that myoActivation, as an adjuvant to interdisciplinary care, is a feasible therapeutic strategy for appropriately selected pediatric patients with myofascial dysfunction and CLBP.

myoActivation has several distinct differences to other needling modalities, such as intramuscular stimulation (IMS) or acupuncture. myoActivation targets active muscular trigger points, fascia in tension, and scars, not just muscles. The stimulus for myoActivation differs significantly due to needle type, insertion techniques, and tissue responses compared to other modalities. myoActivation uses cutting tip needles, which leads to a minimally traumatic stimulus that may trigger a spinal reflex, inducing muscle relaxation. myoActivation needles incorporate a syringe filled with saline but devoid of any pharmacologically active chemicals and do not utilize electrical stimulation. myoActivation and IMS both target myofascial or muscular trigger points [20,21], but the fine gauge hypodermic needles used for myoActivation are quickly inserted and withdrawn, not left in place. IMS and acupuncture may have in-situ needles in the body for 10-45 minutes and may be manipulated to stimulate muscle twitching and promote healing [22,23]. myoActivation principles dictate that the site of perceived pain is often not the true source of pain. For IMS, the site of pain is often the area targeted for treatment.

After remedial causes for CLBP were excluded for patients in this series, myofascial dysfunction was determined to be a contributing component. The myoActivation assessment identifies myofascial sources of pain for all patients by considering each patient’s lifetime trauma, the mechanisms of any identified injuries, postural observations, and systematized BASE movement tests. This provides some diagnostic certainty and hope for targeted therapy especially when the classic medical examination revealed a normal neuromuscular examination. myoActivation examination is not only useful to diagnose myofascial dysfunction but also to show changes with subsequent myoActivation examinations.

To recognize myofascial dysfunction, it is important to understand that many factors contribute to its development, such as genetics, nutrition, immune function, lifestyle, environmental factors cultural influences, ergonomic factors (e.g., overuse athletic activities, abnormal posture), structural factors (e.g., spondylosis, scoliosis, osteoarthritis), and lifetime physical impacts to the body (medical conditions, fractures, injuries and surgeries) [24]. When the skin is injured, the healing process leads to the formation of scar tissue. Remodeling of the scar and the deposition of type 1 collagen increases the strength of the tissue but decreases its elasticity. The subsequent reduced mobility and flexibility impacts not only the skin but also the underlying fascia and muscle. Scar tissue is associated with increased nerve density, neuropeptides, and nociceptors in scarred tissues. Scars were determined to be a contributing factor for all pain profiles within this case series: each patient had scars released, even in the two patients who did not self-report any scars. Scars are often overlooked but play a crucial role in the development and perpetuation of MFD&P as their presence can have significant biomechanical consequences by restricting the flexibility of the scarred skin and the underlying soft tissues [15]. When scars are released, even at sites distant from the area of complaint, the reduction of scar-related tension can improve pain, flexibility, and range of motion [15,17,18].

The fascial system is defined as “a three-dimensional continuum of soft collagen containing loose and dense fibrous connective tissue that permeates the body. It incorporates elements such as adipose tissue, adventitia, neurovascular sheaths, aponeurosis, deep and superficial fascia, epineurium, joint capsules, ligaments, membranes, meninges, myofascial expansions, periostea, retinacula, septa, tendons, visceral fascia, and all the intramuscular and intermuscular connective tissues including endo-/peri-/epi-mysium. The fascial system surrounds, interweaves between, and interpenetrates all organs, muscles, bones, and nerve fibers, endowing the body with a functional structure and providing an environment that enables all body systems to operate in an integrated manner" [25]. As the fascia interweaves and envelopes every structure within the body, it creates structural continuity that provides form and function to the human body [26,27]. It varies in thickness, function, composition, and direction depending on its location and purpose [26]. It is innervated and has proprioceptive and nociceptive properties [27,28]. Injury reduces the flexibility of the fascia. The subsequent defective fascial sliding generates anomalous tension, disorders of intermuscular interaction, and active trigger points in muscles leading to progressive immobility and pain [29]. Half of the patients in this study received fascial release at the iliac crest, further exemplifying how myofascial tissues at and around the pelvis can play an important role in CLBP, this is supported by other work focussing on the psoas muscle fascia [30] and the thoracolumbar and lumbar fascia [31,32].

For patients in this series, the myofascial tissues around the pelvis were important. The most activated muscles in our case series were the iliopsoas, abdominal wall muscles, and paraspinals. Previous studies have outlined the significance of the pelvic muscles in low back pain presentations [33,34] and intramuscular stimulation has been shown to be beneficial for CLBP [35]. Our patients in this series showed improvements in pain, not just from release around the pelvic muscles but also at peripheral or distant sites including the release of fascia and scars (i.e., the release of knee scars). The muscles, fascia, and skin are intricately connected within the myofascial system where myofascial release results in improvement of pain, flexibility, and range of motion [18]. These results strengthen the myoActivation principle that the site of perceived pain is often NOT the true source of pain.

Limitations

The small sample size restricts the ability to generalize these findings to a broader population. Our participants were all female patients, which introduces a gender bias, limiting the applicability of these results to male patients. The patients were treated by a single myoActivation clinician, which may influence the reproducibility of outcomes. The myoActivation process is structured but myoActivation clinician experience and learning have benefited from insights gained from past patients; therefore, the impact of myoActivation may have varied from the beginning of the study period compared to the end of the study. There was a relatively long time in CPS care (admission to discharge), as well as the time between the final myoActivation session and CPS discharge, which may be explained by the concurrence of the COVID-19 pandemic and its impact on in-person assessments at the time. Another contributing factor is the fear that pain will return, especially after so many years of lived experience with chronic pain: patients may request a long follow-up time to be sure they continue to feel better before they are finally discharged.

## Conclusions

This case series provides preliminary evidence that myoActivation may be a clinically useful non-pharmacological tool in the assessment and management of appropriately selected adolescents with MFD&P and CLBP. myoActivation assessment can help diagnose MFD&P, and myoActivation treatment can help adolescents move from a pain-centered life to a function-centered life, even after years of pain. Myofascial dysfunction should be considered as a diagnostic category, not just for patients with CLBP but for any patient with a chronic pain presentation. Prospective longitudinal research is required to establish robust evidence confirming the clinical efficacy of this approach as a component of interdisciplinary care.
